# A phase 1 study of the pharmacokinetics of nucleoside analog trifluridine and thymidine phosphorylase inhibitor tipiracil (components of TAS-102) vs trifluridine alone

**DOI:** 10.1007/s10637-016-0409-9

**Published:** 2017-01-23

**Authors:** James M. Cleary, Lee S. Rosen, Kenichiro Yoshida, Drew Rasco, Geoffrey I. Shapiro, Weijing Sun

**Affiliations:** 1 Dana-Farber Cancer Institute, 450 Brookline Avenue, Boston, MA 02215 USA; 2 University of California, Los Angeles, 2020 Santa Monica Blvd., Ste. 600, Santa Monica, CA 90404 USA; 3 Taiho Oncology, Inc., 101 Carnegie Center, Suite 101, Princeton, NJ 08540 USA; 4 South Texas Accelerated Research Therapeutics, 4319 Medical Dr., Ste. 205, San Antonio, TX 78229 USA; 5 University of Pittsburgh Cancer Institute/UPMC, 5150 Centre Avenue, Fifth Floor, Pittsburgh, PA 15232 USA

**Keywords:** Advanced solid tumors, Fluoropyrimidine, Pharmacokinetics, Trifluridine/tipiracil, Trifluridine

## Abstract

*Background* Trifluridine, a thymidine-based chemotherapeutic, has limited bioavailability after clinical administration as it is rapidly degraded via thymidine phosphorylase. An oral combination tablet combines trifluridine with a potent thymidine phosphorylase inhibitor, tipiracil hydrochloride. This study’s objective was to evaluate whether trifluridine/tipiracil (TAS-102) administration increases trifluridine exposure vs trifluridine alone. *Methods* This open-label pharmacokinetic study randomly assigned patients with advanced solid tumors into two groups. On the morning of day 1, one group received a single 35 mg/m^2^ dose of trifluridine/tipiracil and the other group received a single 35-mg/m^2^ dose of trifluridine. Both groups received trifluridine/tipiracil 35 mg/m^2^ on the evening of day 1, then twice daily on days 2–5 and 8–12 in a 28-day cycle. *Results* Twenty patients received an initial one-time dose of trifluridine alone and 19 other patients received an initial dose of trifluridine/tipiracil. Trifluridine area under the curve (AUC_0-last_) and maximum observed plasma concentrations (C_max_) were approximately 37- and 22-fold higher, respectively, with trifluridine/tipiracil vs trifluridine alone. Plasma concentrations of the major metabolite of trifluridine were lower following the administration of trifluridine/tipiracil vs trifluridine alone. *Conclusion* Tipiracil administered in combination with trifluridine significantly increased exposure to trifluridine compared with trifluridine alone.

## Introduction

Fluoropyrimidines have been widely used for decades for the treatment of multiple neoplasms [1]. In the early 1960s, a potent fluoropyrimidine, trifluridine, was developed [2]. Trifluridine is rapidly degraded to an inactive metabolite, 5-trifluoromethyl-2,4(1*H*,3*H*)-pyrimidinedione (FTY), by thymidine phosphorylase, an enzyme found in the gastrointestinal tract, liver, and tumor tissue [3, 4]. A consequence of trifluridine’s rapid degradation by thymidine phosphorylase is that trifluridine has poor bioavailability [5]. While intravenous trifluridine did have some antitumor activity in early-phase trials, further development of single-agent trifluridine was discontinued because its suboptimal pharmacokinetics (PK) required dosing schedules that were infeasible [5, 6].

The development of a potent and specific chemical inhibitor of thymidine phosphorylase, tipiracil hydrochloride, created an opportunity to improve the PK properties of trifluridine [3]. The coadministration of oral trifluridine and tipiracil in mice and monkeys led to an increased bioavailability of trifluridine [3, 7]. Compared with trifluridine alone, the oral coadministration of trifluridine and tipiracil led to a marked increase in the trifluridine area under the concentration–time curve (AUC) and maximum observed plasma concentration (C_max_) [3].

Trifluridine/tipiracil (TAS-102) is a new orally active antineoplastic agent comprised of trifluridine and tipiracil in a molar ratio of 1:0.5. In Japanese phase 1 and 2 studies in solid tumors, trifluridine/tipiracil was well tolerated, with the primary toxicity being myelosuppression [8–11]. The global phase 3 RECOURSE trial demonstrated that trifluridine/tipiracil is efficacious in refractory metastatic colorectal cancer. The RECOURSE trial showed that, compared with a placebo control, trifluridine/tipiracil increased overall survival in patients with refractory metastatic colorectal cancer by 1.8 months [12]. The results of the RECOURSE trial led the US Food and Drug Administration (FDA) to approve trifluridine/tipiracil for patients with refractory metastatic colorectal cancer.

The objective of the current study was to demonstrate the ability of tipiracil, when administered in combination with trifluridine as trifluridine/tipiracil, to increase the exposure to trifluridine in patients with advanced solid tumors. Exposure to trifluridine was compared after administration of equivalent trifluridine doses either in the presence of tipiracil (as trifluridine/tipiracil) or in the absence of tipiracil (as trifluridine alone).

## Materials and methods

### Study design

**Fig. 1:**
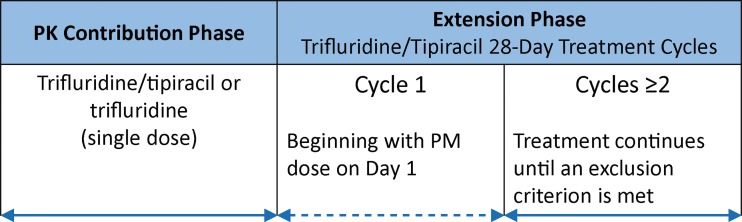
Study design. PK, pharmacokinetics

This was a phase 1, open-label, randomised, parallel, two-group study conducted in four centers in the United States from May 2013 to January 2014. The study was carried out in two stages (single-dose and multiple-dose) using a 28-day treatment cycle (Fig. 1).

### Study population

Men and women aged 18 years or older, with histologically or cytologically confirmed advanced solid tumors for which no standard therapy existed, were selected for study. This study was conducted in accordance with the ethical principles in the Declaration of Helsinki (2008), the US Code of Federal Regulations (Title 21, 312.50 through 312.70), the International Council for Harmonisation Tripartite Guidelines for Good Clinical Practice, and local and national laws and regulations governing the use of investigational therapeutic agents. The study protocol and other relevant documents received approval from the Institutional Review Board/Independent Ethics Committee prior to patient enrollment. Written informed consent was obtained from each patient. The study is registered on ClinicalTrials.gov: NCT01867866.

Patients were required to have Eastern Cooperative Oncology Group (ECOG) performance status 0 or 1 on day 1 of cycle 1 as well as being able to take medications orally. The eligibility criteria also required a serum creatinine ≤1.5 mg/dL, serum bilirubin ≤1.5 × the upper limit of normal (ULN), and adequate function of the bone marrow (absolute neutrophil count ≥1500/μL; platelets ≥100,000/mm^3^; hemoglobin ≥9.0 g/dL).

Major exclusion criteria included treatment with anticancer therapy within the prior 3 weeks (mitomycin within the prior 5 weeks), investigational agent received either concurrently or within the last 30 days or five half-lives (whichever was shorter), current enrolment in another interventional clinical study, serious illness or medical condition, and unresolved toxicity of National Cancer Institute Common Terminology Criteria for Adverse Events (NCI CTCAE) Grade ≥ 2 attributed to prior therapies.

### Treatment

Patients were stratified into two groups according to body surface area (<1.8 m^2^; ≥1.8 m^2^) and randomized to receive a single dose of trifluridine/tipiracil (group 1) or trifluridine alone (group 2).

#### Single dose

The single-dose stage of the study was conducted on the morning of day 1 of cycle 1. After an overnight fast of at least 8 h and within 30 min after completion of a standardized, high-fat, high-calorie breakfast, a dose of trifluridine/tipiracil (trifluridine:tipiracil molar and weight ratios of 1:0.5 and 1:0.471, respectively) or trifluridine alone was administered. On the morning of day 1, cycle 1, patients in group 1 received a single oral dose of trifluridine/tipiracil 35 mg/m^2^, while patients in group 2 received a single oral dose of trifluridine 35 mg/m^2^. Blood samples were collected from all patients on day 1 at the following times: 0 (predose) and 15 min, 30 min, 1 h, 1 h 30 min, 2, 3, 4, 6, 8, 10, and 12 h postdose.

#### Multiple dose

All patients (from groups 1 and 2 of the single-dose stage) received trifluridine/tipiracil in the multiple-dose stage. The multiple-dose stage of the study began with the evening dose of trifluridine/tipiracil on day 1 of cycle 1 after collection of the 12-h postdose PK blood sample in the single-dose stage. Trifluridine/tipiracil 35 mg/m^2^/dose was then administered orally twice daily on days 2–5 of cycle 1, with doses administered within 1 h after completion of the morning (am dose) and evening (pm dose) meals. This was followed by a recovery period from day 6 to day 7. Trifluridine/tipiracil was again administered orally twice daily on days 8–12, with the last dose administered in the evening of day 12. Blood samples were collected on day 12 of cycle 1 at the following times: 0 (am predose) and 30 min, 1, 2, 4, 8, and 12 h postdose. Day 12 was followed by a recovery period from day 13 to day 28.

All patients continued to receive the 28-day cycle of trifluridine/tipiracil 35 mg/m^2^ until disease progression, intolerable toxicity, or need for more than three dose reductions of trifluridine/tipiracil due to an unacceptable adverse event (AE). Blood samples were collected on day 12 of cycles 2 and 3 at the following times: 0 (am predose), and 30 min, 1, 2, 4, 8, and 12 h postdose. Efficacy and safety assessments were performed during the multiple-dose stage of the study.

### Evaluation parameters

To assess treatment compliance, the number of tablets dispensed and returned by the patient was recorded for each treatment cycle. Any dose reductions or interruptions were recorded along with the reasons for those actions.

#### Pharmacokinetics

PK parameters for trifluridine, FTY, and tipiracil in plasma following administration of a single dose of trifluridine/tipiracil or trifluridine alone were determined using standard, noncompartmental methods. The primary endpoints for comparison of trifluridine/tipiracil and trifluridine alone (single-dose) were AUC from hour 0 to the time of last measurable plasma concentration (AUC_0-last_) (after the morning dose) for trifluridine estimated by the linear trapezoidal rule and C_max_ of trifluridine after the morning dose.

The secondary endpoints for single-dose administration were PK parameters for trifluridine, FTY, and tipiracil, including AUC from time zero to infinity (AUC_0-inf_), time of maximum observed plasma concentration (T_max_), and terminal half-life (T_½_). Secondary endpoints for multiple-dose administration were PK parameters for trifluridine, FTY, and tipiracil. AUC_0-last_, C_max_, T_max_, and T_½_ for those compounds and were determined on day 12 of cycles 1, 2, and 3.

#### Efficacy

For patients who were continuing to receive treatment at the time of the data cutoff, data were collected for all completed cycles prior to and including the cutoff date. Tumor assessments were performed throughout the study based on review of computed tomography (CT) scans and following Response Evaluation Criteria in Solid Tumors (RECIST) version 1.1, 2009. CT scans of the chest, abdomen, and pelvis (as clinically indicated) were performed at the end of every 8 weeks through cycle 6. After cycle 6, the follow-up tumor assessment schedule could be adjusted to conform to the site standard of care, provided that follow-up scans were performed at least every 12 weeks. Disease control was defined as having complete response, partial response, or stable disease. For a patient to be categorized as having “stable disease,” the patient had to maintain stable disease for at least 6 weeks from the start of treatment.

#### Safety

Standard safety monitoring and grading were performed using NCI CTCAE version 4.03. The evaluation of safety was based on the incidence, severity, and causality of adverse events (AEs) and serious AEs and other safety assessments, including physical examination, vital signs, ECOG performance status, 12-lead electrocardiogram, and clinical laboratory evaluations. Safety data were summarized descriptively.

### Statistical methods

#### Sample size

The investigators who designed this study planned to enroll approximately 40 patients. Assuming an attrition rate of 20% for dropouts and patients for whom PK data were not available or able to be evaluated, a total of 20 patients per treatment group were to be enrolled in the single-dose stage to ensure that at least 16 patients in each group completed this stage of the study. This would provide 80% power to detect a 1.67-fold change (ratio of the geometric means of AUCs for trifluridine as trifluridine/tipiracil and trifluridine alone), that is, a 67% increase in the trifluridine/tipiracil arm. This calculation was based on a two-group t-test, assuming a log-normal distribution, a coefficient of variation (CV) of 50%, one interim analysis, and a two-sided overall study alpha of 5%.

No prior clinical information existed for the expected increase in trifluridine AUC with trifluridine/tipiracil relative to trifluridine alone. The targeted effect of 67% was chosen so that the minimum detectable difference was at least as large as the inherent variability in trifluridine AUC (CV of 50%). At least six patients with evaluable multiple-dose PK profiles for all three cycles and no dose reductions for the first three cycles of treatment in the multiple-dose stage were required to satisfy the additional study objective to investigate multiple-dose PK of trifluridine/tipiracil.

#### Pharmacokinetics

The PK parameters were compared for trifluridine/tipiracil and trifluridine alone using a t-test based on the log-transformed PK parameters. Ninety-five percent CIs were derived for the differences in the log-transformed means. Point estimates and confidence limits were exponentiated after the analysis and presented as ratios. PK parameters for trifluridine, FTY, and tipiracil in plasma following administration of multiple doses of trifluridine/tipiracil (day 12 of cycles 1, 2, and 3) were summarized descriptively.

## Results

### Patient disposition

A total of 44 patients were enrolled, randomized, and treated, with 22 patients in the single-dose group of trifluridine/tipiracil (group 1) and 22 patients in the single-dose group of trifluridine (group 2). Of the patients who participated in the single-dose stage of the study, 39 (88.6%) were evaluable. Three patients in group 1 were excluded for the following reasons: incorrect dose, significant inclusion criteria violation, and fasting conditions not met (one patient each). Two patients in group 2 were excluded due to an error in collecting blood samples or missing samples/assays (one patient each). A total of 39 patients were evaluable as a single-dose PK population, with 19 patients in the trifluridine/tipiracil group and 20 patients in the trifluridine alone group.

Demographic and baseline characteristics were similar for patients in group 1 and group 2 in the single-dose PK population (Table 1) as well as in the safety population. The study population consisted of 22 men and 22 women with a mean age of 57.0 years; 90.9% were white and 75% had an ECOG performance status of 1. The majority (61.4%) of patients had colon cancer; 68.2% had received ≥4 prior chemotherapy regimens, indicating that they were heavily pretreated.

**Table 1:** Demographic and baseline characteristics, single-dose stage

Parameter	Single-Dose PK Population (*N* = 39)			Safety Population (*N* = 44)
	Trifluridine/tipiracil (*n* = 19)	Trifluridine (*n* = 20)	All (*N* = 39)	
Mean age (± SD), y	56.2 (11.69)	57.5 (7.58)	56.9 (9.69)	57.0 (10.04)
Gender, n (%)				
Male	10 (52.6)	11 (55.0)	21 (53.8)	22 (50.0)
Female	9 (47.4)	9 (45.0)	18 (46.2)	22 (50.0)
Race, n (%)				
White	17 (89.5)	19 (95.0)	36 (92.3)	40 (90.9)
Black/African heritage	1 (5.3)	1 (5.0)	2 (5.1)	3 (6.8)
Asian	1 (5.3)	0	1 (2.6)	1 (2.3)
Mean weight (± SD)^a^, kg	81.8 (18.07)	73.7 (15.54)	77.7 (17.09)	77.3 (17.88)
BSA, mean (± SD), m^2^	1.95 (0.244)	1.82 (0.214)	1.88 (0.234)	1.88 (0.246)
Stratification BSA^a^, n (%)				
< 1.8 m^2^	5 (26.3)	8 (40.0)	13 (33.3)	16 (36.4)
≥ 1.8 m^2^	14 (73.7)	12 (60.0)	26 (66.7)	28 (63.6)
ECOG performance status, n (%)				
0	6 (31.6)	3 (15.0)	9 (23.1)	11 (25.0)
1	13 (68.4)	17 (85.0)	30 (76.9)	33 (75.0)
Prior radiotherapy, n (%)				
Yes	6 (31.6)	12 (60.0)	18 (46.2)	20 (45.5)
No	13 (68.4)	8 (40.0)	21 (53.8)	24 (54.5)
Number of regimens^b^, n (%)				
1–2	3 (15.8)	6 (30.0)	9 (23.1)	10 (22.7)
3	3 (15.8)	1 (5.0)	4 (10.2)	4 (9.1)
≥ 4	13 (68.4)	13 (65.0)	26 (66.7)	30 (68.2)

*BSA* body surface area, *ECOG* Eastern Cooperative Oncology Group, *PK* pharmacokinetics, *SD* standard deviation

^a^Per Interactive Web Response System (height and weight collected at baseline)

^b^Includes all prior systemic therapies (neoadjuvant, adjuvant, metastatic)

Following the initial morning dose on day 1, all patients in both groups (*n* = 44) went on to the multiple-dose stage of the study. Six patients who received at least one cycle of treatment were excluded from the analysis due to missing PK data (six patients) and/or dosing deviation (two patients). A total of 38 patients were evaluable for PK analysis for at least one cycle; of these, nine patients were evaluable for PK analysis at cycle 3. The remainder of the patients discontinued treatment or had a dose reduction prior to the completion of cycle 3 PK assessment.

As of the data cutoff date (January 21, 2014), 36 (81.8%) patients had discontinued study treatment. The most frequent reason for discontinuing treatment was radiographic evidence of disease progression (36.4%), with clinical disease progression (20.5%) being the second most frequent cause. Patient cancer types included colon (61.4%), pancreas (11.4%), rectal (4.5%), head/neck (4.5%), esophagus (2.3%), ovary (2.3%), and others (13.6%).

### Pharmacokinetics

#### Single dose

**Fig. 2:**
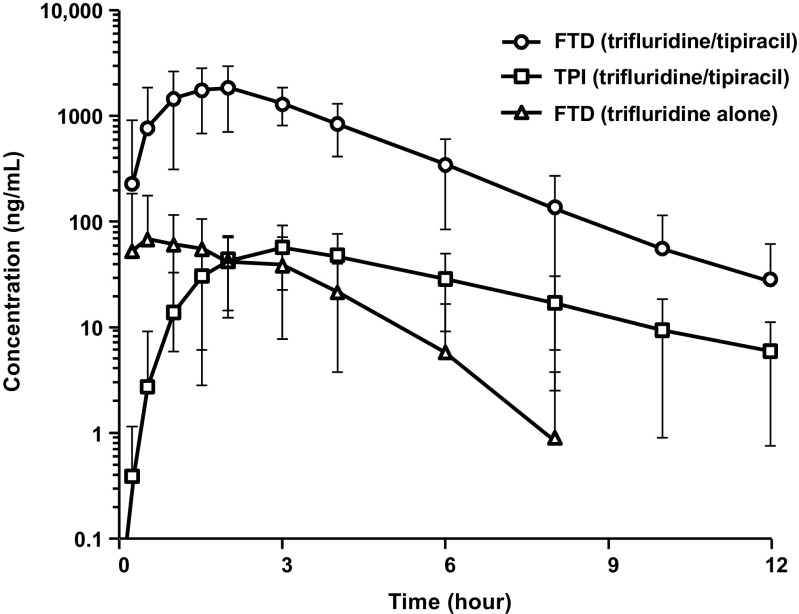
Single-dose PK studies. For this analysis, patients received either a single oral dose of trifluridine/tipiracil at 35 mg/m^2^ (dose rounded to the nearest 5 mg) or a single oral dose of trifluridine at 35 mg/m^2^. Blood samples were collected from all patients at the following times: 0 (predose) and 15 min, 30 min, 1 h, 1 h 30 min, 2, 3, 4, 6, 8, 10, and 12 h postdose. FTD, trifluridine; PK, pharmacokinetic; TPI, tipiracil

Administration of trifluridine/tipiracil resulted in a significant increase in exposure to trifluridine compared with administration of trifluridine alone (Fig. 2). Based on the ratio of the geometric mean estimates, trifluridine AUC_0-last_ was approximately 37-fold higher following administration of trifluridine/tipiracil than following administration of trifluridine alone (Table 2). In addition, trifluridine C_max_ was approximately 22-fold higher and trifluridine AUC_0-inf_ was 27-fold higher for trifluridine/tipiracil compared with trifluridine alone (Table 2).

**Table 2:** Single-dose PK of trifluridine/tipiracil and trifluridine

Parameter	Trifluridine/tipiracil		Trifluridine		Ratio of Geometric Mean ([Trifluridine/tipiracil]/Trifluridine)	
	N	Geometric Mean	N	Geometric Mean	Estimate	95% CI
Trifluridine						
AUC_0-last_ (ng•h/mL)	19	6618	20	176	37.55	27.56–51.15
C_max_ (ng/mL)	19	2155	20	96	22.39	14.19–35.34
AUC_0-inf_ (ng•h/mL)	19	6694	10^a^	248	27.00	19.56–37.27
FTY						
AUC_0-last_ (ng•h/mL)	19	3232	20	4122	0.78	0.65–0.94
C_max_ (ng/mL)	19	737	20	1104	0.67	0.54–0.82
AUC_0-inf_ (ng•h/mL)	19	3320	20	4179	0.79	0.66–0.96

*AUC*_*0-last*_ area under the curve from hour 0 to the last measurable plasma concentration, *AUC*_*0-inf*_ area under the curve from hour 0 to infinity, *C*_*max*_ maximum observed plasma concentration, *FTY* 5-trifluoromethyluracil, *PK* pharmacokinetics

^a^Due to low and fluctuating plasma trifluridine concentrations after administration of trifluridine alone, AUC_0-inf_ could only be determined for 10 patients

As expected, plasma concentrations of FTY (inactive metabolite) were lower following administration of trifluridine/tipiracil compared with trifluridine alone due to extensive metabolism of trifluridine when administered alone. The ratio of the geometric mean estimates was less than 1 for AUC_0-last_ (0.78), C_max_ (0.67), and AUC_0-inf_ (0.79) (Table 2).

The mean and geometric mean C_max_ values for trifluridine after administration of trifluridine alone were lower (138 and 96 ng/mL, respectively) than after administration of trifluridine/tipiracil (2381 and 2155 ng/mL, respectively) (Tables 2 and 3). For trifluridine C_max_ calculations, the CV was higher after administration of trifluridine alone than after administration of trifluridine/tipiracil (92% vs 44%, respectively) (Table 3). The lower and more variable plasma concentrations of trifluridine are consistent with the poor bioavailability of trifluridine after oral administration of trifluridine alone.

**Table 3:** Descriptive statistics for trifluridine, FTY, and tipiracil PK parameters after ≥1 cycle of trifluridine/tipiracil

Parameter	Trifluridine/tipiracilsingle dose	Trifluridine alone single dose	Trifluridine/tipiracil Multiple Dose (≥1 cycle)		
	Cycle 1, Day 1 (*N* = 19)	Cycle 1, Day 1 (*N* = 20)	Cycle 1, Day 12 (*N* = 34)	Cycle 2, Day 12 (*N* = 25)	Cycle 3, Day 12 (*N* = 9)
Trifluridine					
AUC_0-last_ (ng•h/mL)					
Mean	7045	200	23,697	25,056	26,696
SD	2411	96	7419	10,586	9219
CV	34%	48%	31%	42%	35%
C_max_ (ng/mL)					
Mean	2381	138	4857	5458	5297
SD	1048	127	1930	2269	2291
CV	44%	92%	40%	42%	43%
T_max_ (h)					
Median	1.50	1.50	1.97	2.00	2.00
Min, Max	0.53, 4.00	0.25, 6.00	0.50, 8.00	0.50, 4.00	1.00, 4.00
T_1/2_ (h)					
N^a^	19	10	26	19	5
Mean	1.42	1.14	2.07	2.10	2.55
SD	0.42	0.55	0.43	0.50	0.79
CV	30%	48%	21%	24%	31%
FTY					
AUC_0-last_ (ng•h/mL)					
Mean	3344	4281	5206	5736	5832
SD	897	1132	2055	2345	1938
CV	27%	26%	39%	41%	33%
C_max_ (ng/mL)					
Mean	765	1169	679	754	783
SD	201	402	200	205	220
CV	26%	34%	29%	27%	28%
T_max_ (h)					
Median	3.00	2.00	2.00	2.00	3.93
Min, Max	1.00, 6.08	0.30, 6.00	0.50, 8.00	1.00, 8.00	1.03, 4.00
T_1/2_ (h)					
N^a^	19	20	9	6	0
Mean	1.76	1.28	4.51	3.76	NA
SD	0.38	0.33	0.53	0.59	NA
CV	21%	26%	12%	16%	NA
Tipiracil					
AUC_0-last_ (ng•h/mL)					
Mean	301	–	372	333	299
SD	127	–	135	124	92
CV	42%	–	36%	37%	31%
C_max_ (ng/mL)					
Mean	69	–	69	66	54
SD	30	–	27	25	17
CV	43%	–	40%	39%	32%
T_max_ (h)					
Median	3.00	–	2.01	3.25	4.00
Min, Max	1.02, 8.00	–	1.00, 8.03	1.00, 8.00	1.97, 4.08
T_1/2_ (h)^a^					
N^a^	16	–	19	12	2
Mean	2.10	–	2.40	2.51	2.31
SD	0.47	–	0.59	0.69	1.03
CV	22%	–	24%	27%	44%

*AUC*_*0-last*_ area under the curve from hour 0 to the last measurable plasma concentration, *C*_*max*_ maximum observed plasma concentration, *CV* coefficient of variation, *FTY* 5-trifluoromethyluracil, *NA* not available, *Max* maximum, *Min* minimum, *PK* pharmacokinetics, *SD* standard deviation, *T*_*1/2*_ terminal half-life, *T*_*max*_ time of maximum observed plasma concentration

^a^Due to fewer sampling time points on day 12 (30 min, 1, 2, 4, 8, and 12 h postdose), half-life could not be calculated for some patients

In comparing trifluridine/tipiracil with trifluridine alone administration, trifluridine/tipiracil had a higher trifluridine geometric mean AUC_0-last_ (6618 vs 176 ng•h/mL, respectively), similar trifluridine median T_max_ (1.50 vs 1.50 h, respectively), and similar trifluridine mean T_½_ time (1.42 vs 1.14 h, respectively) (Tables 2 and 3). This suggests that bioavailability was the primary difference between trifluridine/tipiracil and trifluridine alone.

#### Multiple dose

Descriptive statistics for trifluridine plasma PK parameters following single- and multiple-dose trifluridine/tipiracil administration show that the mean trifluridine AUC_0-last_ was approximately 3-fold higher than after single-dose trifluridine/tipiracil administration (*n* = 19) and that mean C_max_ was approximately 2-fold higher. The AUC_0-last_ for FTY was also increased after multiple dosing of trifluridine/tipiracil compared with a single dose; however, C_max_ values for FTY were similar after single and multiple dosing. For tipiracil, AUC_0-last_ and C_max_ were similar after single- and multiple-dose administration of trifluridine/tipiracil (Table 3).

### Safety

#### Dose reductions and delays

Across all cycles, 17 of 44 (38.6%) patients received concomitant granulocyte colony-stimulating factor and 39 of 44 (88.6%) patients received ≥80% of their target cycle dose.

Thirty-eight of 44 (86.4%) patients maintained the starting dose of 35 mg/m^2^ twice daily throughout their participation in the study. Six (13.6%) patients had a single dose reduction to 30 mg/m^2^ twice daily at the start of cycle 2 or 3. For these six patients, dose reductions were due to neutropenia (four patients), febrile neutropenia (one patient), and anemia (one patient). Of the 36 patients who initiated at least two cycles of treatment, eight (22.2%) experienced at least one cycle initiation delay of ≥8 days. Eight of all 61 (13.1%) cycles administered were delayed by ≥8 days. Of the eight patients with a delay of ≥8 days in cycle initiation, the reasons were Grade 3/4 neutropenia (five patients), Grade 2 neutropenia (two patients), and Grade 4 febrile neutropenia (one patient). As of the data cutoff date of the extension stage of the study, 44 patients had initiated at least one cycle of trifluridine/tipiracil treatment. The mean number of cycles initiated was 2.4 and the mean number of weeks followed was 7.7 (range 1–6).

AEs were reported for 43 of 44 patients treated; most patients (88.6%) had AEs that were considered treatment-related. The most frequently reported treatment-related AEs were nausea (47.7%), fatigue (31.8%), anemia (27.3%), decreased neutrophil count (25.0%), diarrhea (22.7%), vomiting (22.7%), and decreased white blood cell count (18.2%). The most common Grade ≥ 3 treatment-related AEs (reported for ≥10% of patients) were anemia (18.2%), neutropenia (13.6%), and leukopenia (13.6%). Five patients died as of the cutoff date; three deaths were attributed to clinical disease progression, one to radiologic disease progression, and one to upper gastrointestinal haemorrhage and hemorrhagic shock, which was not considered treatment-related. Six patients experienced a serious AE, which was considered treatment-related, the most frequent of which were cytopenias. Two patients experienced serious AEs classified as decreased appetite and dehydration.

### Efficacy

At the time of data cutoff, there were no partial or complete responses observed; 23 patients had stable disease for ≥6 weeks (according to standard RECIST criteria), and 18 patients had progressive disease. The rate of disease control was 52%.

## Discussion

Previous efforts to develop trifluridine as a therapeutic agent were hampered by its poor bioavailability, which is due to its rapid degradation via thymidine phosphorylase. The development of the thymidine phosphorylase inhibitor, tipiracil, created an opportunity to utilize trifluridine in the clinic. TAS-102, a combination tablet of trifluridine and tipiracil, was developed based on the hypothesis that inhibition of thymidine phosphorylase would increase the bioavailability of trifluridine. The results of this randomized PK study are proof of concept that inhibition of thymidine phosphorylase with tipiracil leads to a substantially increased exposure to trifluridine. Compared with trifluridine alone, the combination of tipiracil and trifluridine resulted in a 37-fold increase in AUC_0-last_ and 22-fold increase in C_max_ (Table 2). Importantly, these results demonstrate the pharmacologic feasibility of utilizing an oral combination of trifluridine and tipiracil. This is further supported by the placebo-controlled, phase 3 RECOURSE trial, which found that trifluridine/tipiracil improved survival in patients with refractory colorectal cancer [12].

Preclinical pharmacology studies similarly found that oral coadministration of trifluridine and tipiracil markedly increases the C_max_ and AUC of trifluridine. In monkeys, the oral coadministration of equimolar trifluridine and tipiracil led to a 70-fold increase in C_max_ and approximately a 100-fold increase in AUC [3]. The results obtained in the present clinical study were similar to those observed in the preclinical studies on monkeys. Furthermore, the results of this study demonstrate the poor bioavailability of trifluridine when administered alone, which is also consistent with the results of previous preclinical and clinical trials performed on trifluridine [5]. As shown in Tables 2 and 3, the C_max_ of trifluridine after administration of trifluridine alone was significantly lower than that after trifluridine/tipiracil (96 ng/mL for trifluridine alone vs 2155 ng/mL for trifluridine/tipiracil). In addition to trifluridine alone having poor bioavailability, it also had more variable plasma concentrations of trifluridine. Single-agent trifluridine’s C_max_ CV (CV = 92%; range: 25–504 ng/mL) was approximately double that observed after the administration of trifluridine/tipiracil (CV = 44%; range: 979–4190 ng/mL). The lower and more variable plasma concentrations of trifluridine when given alone confirmed that trifluridine cannot be effectively administered orally as an anticancer therapy without being combined with tipiracil (Fig. 2).

The 3-fold higher mean trifluridine AUC_0-last_ after multiple doses of trifluridine/tipiracil is consistent with a previous study in Japanese patients, in which the accumulation ratio for trifluridine AUC was 2.4 (at the same dosage and dosing regimen) (Table 3) [10]. Although the mechanism has not been identified, the accumulation of trifluridine after repeated administration does not appear to be a safety or efficacy risk of trifluridine/tipiracil because the accumulation of trifluridine AUC is not dose-dependent [10]. Furthermore, the variability for the accumulation ratio of trifluridine is relatively small, as it ranged from 2- to 4.5-fold (Table 3). Multiple-dose PK analyses show that accumulation of trifluridine is limited to the first 28-day treatment cycle, with no further accumulation over subsequent cycles, up to cycle 3 in this study. Similar results were observed for FTY and tipiracil.

In this study, which utilized the same dosage regimen as in the RECOURSE trial, trifluridine/tipiracil was found to be generally well tolerated in heavily pretreated patients with solid tumors. Similar to the safety results of the RECOURSE trial, the major toxicity in this study was myelosuppression. In terms of antitumor efficacy, no objective responses to trifluridine/tipiracil were observed in this trial, which is consistent with the observations of the RECOURSE trial. While trifluridine/tipiracil treatment led to a 1.8-month improvement in survival compared with placebo, the RECOURSE trial demonstrated that the response rate to trifluridine/tipiracil in patients with refractory metastatic colorectal cancer was only 1.6%. In RECOURSE, disease control was achieved in 44% patients in the trifluridine/tipiracil group compared to 16% patients in the placebo group [12]. Consistent with this, disease control was observed in 52% of patients in our trial.

In conclusion, exposure to trifluridine is significantly increased when it is administered in combination with tipiracil. This demonstrates the essential contribution of tipiracil and the rationale for its combination with trifluridine in the form of trifluridine/tipiracil. Similar to the observations in other trials, trifluridine/tipiracil was well tolerated in this study and the major toxicity was myelosuppression. Trifluridine/tipiracil is now approved in Japan, Europe, and the United States for patients with refractory metastatic colorectal cancer; further studies are exploring its efficacy in other malignancies.
